# Structure–Property Relationships in PDLLA/Silica Hybrid Films: Impact of Grafting and Network Formation on Optical Behavior

**DOI:** 10.3390/polym17233202

**Published:** 2025-11-30

**Authors:** Shuta Hara, Keiya Kawamura, Atsushi Furukawa, Shigeru Shimizu, Hiroki Ikake

**Affiliations:** 1 Department of Material and Life Chemistry, Kanagawa University, 3-6-1, Kanagawa-ku, Yokohama 221-8686, Japan; 2 Department of Materials and Applied Chemistry, College of Science and Technology, Nihon University, 1-8-14 Kandasurugadai, Chiyoda-ku, Tokyo 101-8308, Japan

**Keywords:** sustainable functional materials, optical transparency, polymer-grafted nanoparticles, sol–gel method, small-angle X-ray scattering (SAXS)

## Abstract

Transparent PDLLA/silica hybrid films were prepared via a sol–gel process using organosilane-terminated PDLLA, and two structural motifs—graft-type and 3D-network hybrids—were systematically compared. Dynamic mechanical analysis (DMA) revealed that silica incorporation significantly restricted polymer chain mobility, increasing the onset temperature of the storage modulus from 33.9 °C for neat PDLLA to 41.5 °C and 50.3 °C for the 15 and 20 wt% graft-type hybrids, respectively. Thermogravimetric analysis (TGA) confirmed silica contents of 8.8–18.5 wt% and showed that the 10% weight-loss temperature increased by ~60 °C relative to neat PDLLA, with improvements primarily governed by silica content rather than hybrid topology. Small-angle X-ray scattering (SAXS) demonstrated uniform nanoscale dispersion with inter-domain distances of ~60–65 nm and no domain coarsening; combining these distances with the PDLLA end-to-end distance (R_0_ ≈ 24–30 nm) yielded effective silica domain sizes of 30–35 nm. Porod analysis distinguished diffuse interfaces in graft-type hybrids from more correlated structures in network-type hybrids. Optically, the hybrids maintained high transparency (>90% at 400 nm) up to 18 wt% silica, while the Abbe number increased from 55 (neat PDLLA) to 73 (20 wt%). These findings provide quantitative insight into how nanoscale silica organization dictates thermomechanical, thermal, and optical behavior in PDLLA hybrids, extending the understanding established by earlier studies and supporting the continued development of PDLLA/silica hybrid materials.

## 1. Introduction

The annual corrosion loss of coating materials accounts for nearly 2% of the world’s Gross Domestic Product (GDP) [1], highlighting the need for sustainable and environmentally compatible coating technologies. Polylactic acid (PLA) and its stereoisomers, poly(L-lactic acid) (PLLA) and poly(D,L-lactic acid) (PDLLA), are bio-derived, biocompatible, and biodegradable polymers that have attracted considerable attention for diverse applications [2,3]. In particular, PLLA exhibits good mechanical strength and is widely used in sutures, commodity plastics, and surgical devices [4,5]. Its high degradability also addresses ecological concerns related to plastic waste accumulation. By contrast, racemic PDLLA is amorphous and offers advantages of high optical transparency and faster environmental degradation than PLLA [6]. However, PDLLA suffers from poor thermal resistance and mechanical strength. Enhancing its thermomechanical properties while maintaining transparency could enable the development of highly sustainable coating materials.

Nanofiller reinforcement has been widely explored to improve the properties of PLA and PDLLA. Hybrid composites incorporating carbon nanotubes [7], hydroxyapatite [8], and clay particles [9] have been reported. Among these, silica is particularly attractive due to its low cost, environmental friendliness, and compatibility with PLA [10,11]. Effective reinforcement requires uniform nanoparticle dispersion within the polymer matrix [12]. The sol–gel method provides a powerful approach, enabling in situ formation of well-dispersed nanoparticles with small sizes and high packing densities, thereby minimizing agglomeration [13,14,15,16]. Moreover, polymers functionalized with terminal organosilanes can undergo sol–gel reactions with alkoxides such as tetraethyl orthosilicate (TEOS), simultaneously generating silica nanoparticles and forming three-dimensional networks [17,18]. For example, Mazzocchetti et al. reported telechelic PDLLA–silane oligomers that produced highly filled, transparent silica hybrid films via 3D network formation [19].

Although three-dimensional (3D) networks ensure uniform nanoparticle dispersion, their processability is limited [17,20]. Alternatively, polymer-grafted nanoparticles (PGNs) offer a promising strategy for achieving high nanoparticle dispersion in both solution and melt states [12,21,22,23]. Grafted chains reduce interparticle attractions, suppress aggregation, and facilitate easier processing. Régibeau et al. demonstrated that as little as 1 wt% PDLLA-grafted silica nanoparticles significantly enhanced the mechanical properties of PDLLA films [6]. Unlike 3D networks, PGNs retain fluidity, enabling more efficient molding and processing. For optical applications such as refractive-index tuning, however, high nanoparticle loading is required. Grafted structures can accommodate such high inorganic content while maintaining long-range nanoparticle ordering, potentially improving both mechanical and optical performance [24]. Despite extensive literature on PDLLA–silica composites, most studies have primarily focused on mechanical reinforcement or thermal resistance. In contrast, the combined influence of nanoscale silica dispersion, polymer–silica interfacial structure, and optical behavior has not been systematically investigated. In particular, how graft-type architectures control silica domain size, inter-domain spacing, and chain-mobility suppression—and how these factors influence optical transparency and chromatic-dispersion behavior—remains unclear. This gap is critical because optical functionalities, such as refractive-index control and Abbe-number enhancement [6,25,26] depend sensitively on nanoscale structural uniformity rather than solely on mechanical reinforcement.

In this study, graft-type PDLLA/silica hybrid films were prepared via the sol–gel method and compared with 3D-network hybrids derived from telechelic PDLLA precursors. To elucidate structure–property relationships, thermomechanical behavior was characterized using dynamic mechanical analysis (DMA), while thermogravimetric analysis (TGA) assessed thermal stability. The nanoscale organization of silica domains—including domain size, spacing, and network formation—was probed by small-angle X-ray scattering (SAXS). Optical properties, such as transparency, refractive index, and Abbe number, were then evaluated to determine how nanoscale dispersion influences light propagation through the hybrid films. This integrated approach emphasizes mechanical, thermal, structural, and optical characterization equally, enabling a comprehensive understanding of how graft-type architectures govern both polymer dynamics and photonic behavior.

## 2. Materials and Methods

### 2.1. Materials

Tetrahydrofuran (THF), n-hexane, and 1.0 M hydrochloric acid (HCl) were obtained from Kanto Chemical Co., Ltd. (Tokyo, Japan). 3-Aminopropyltriethoxysilane (APTEOS) and isocyanatopropyltriethoxysilane (IPTEOS) were purchased from Gelest, Inc. (Morrisville, PA, USA). Tetraethyl orthosilicate (TEOS) and PDLLA (molecular weight 75,000–120,000 g/mol) were obtained from Sigma-Aldrich (Burlington, MA, USA).

### 2.2. Synthesis of Mono-Silane-Terminated ET-PDLLA I and II

ET-PDLLA I was synthesized via aminolysis of the ester end groups of PDLLA. Specifically, PDLLA (1.0 g, 0.0133 mmol) was dissolved in 25 mL of dehydrated THF (polymer concentration: 4.0 *w*/*v*%). A tenfold molar excess of APTEOS (0.133 mmol, 29.0 μL) was then added dropwise under a nitrogen atmosphere. The reaction mixture was stirred at 70 °C for 5 h. The crude product was isolated by precipitation into 200 mL of n-hexane, followed by removal of residual solvent under reduced pressure. The resulting ET-PDLLA I was immediately dissolved in THF (25 mL, 4.0 *w*/*v*%) for use in the subsequent sol–gel reaction.

ET-PDLLA II was synthesized through a urethane-forming reaction between the hydroxyl terminal groups of PDLLA and the isocyanate group of isocyanatopropyltriethoxysilane (IPTEOS). ET-PDLLA I (1.0 g) was dissolved in 25 mL of THF (4.0 *w*/*v*%), followed by the addition of IPTEOS (equimolar to APTEOS, 29.0 μL, 0.133 mmol). The mixture was reacted at 60 °C for 24 h under nitrogen. The resulting ET-PDLLA II was purified by precipitation into 200 mL of n-hexane. After evaporation of residual solvent, the product was redissolved in THF (25 mL) for the sol–gel process. Because undesirable crosslinking was observed upon storage, ET-PDLLA II was used immediately after preparation.

### 2.3. Preparation of PDLLA/SiO_2_ Hybrid Films

**Scheme 1:**
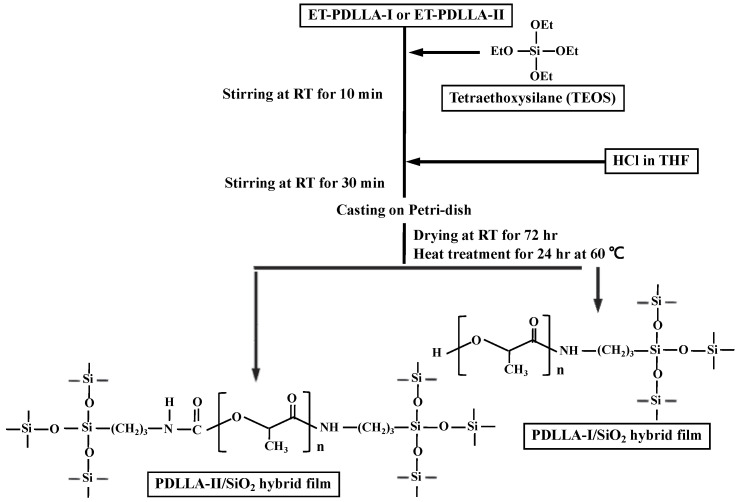
Preparation of the PDLLA/SiO_2_ hybrid materials.

Hybrid films were prepared via a sol–gel reaction between ET-PDLLA I or II and TEOS, catalyzed by HCl, as illustrated in Scheme 1. ET-PDLLA I or II (2.5 g, 0.0333 mmol) was dissolved in 25 mL of THF (polymer concentration: 10.0 *w*/*v*%) at ambient conditions. TEOS was then added dropwise at 15 or 25 wt% relative to ET-PDLLA (0.375 g or 0.625 g, 1.80 or 3.00 mmol). A mixture of THF (282 μL) and 1 M HCl (25 μL) was added slowly to the reaction solution, resulting in a final HCl concentration of approximately 1.0 × 10^−3^ mol/L. The mixture was stirred at 25 °C for 30 min and then cast onto a Petri dish. The films were allowed to dry at room temperature for 72 h, followed by heat treatment at 60 °C for 24 h under an argon atmosphere to yield the PDLLA–I/SiO_2_ and PDLLA–II/SiO_2_ hybrid films.

### 2.4. Characterization

TGA was performed using a TG/DTA-6200 (Hitachi High Technologies, Tokyo, Japan) at a heating rate of 5 °C/min under a nitrogen flow of 200 mL/min to determine the total silica content. Dynamic mechanical analysis (DMA) was conducted with a DMS 6100 (Hitachi High Technologies, Tokyo, Japan). The glass transition temperature (T_g_) and storage modulus (*E*’) of the hybrid films were measured in tension mode at 1 Hz over a temperature range of −10 to 200 °C, with a heating rate of 5 °C/min and a nitrogen flow of 200 mL/min. Small-angle X-ray scattering (SAXS) measurements were carried out using an Anton Paar Kratky compact camera (Anton Paar, Tokyo, Japan) equipped with an 80 µm entrance slit and a 200 µm counter slit. The X-ray voltage and current were 50 kV and 40 mA, respectively. Counting was performed using a proportional counter with a Ni filter and a pulse height analyzer to select only CuKα radiation (*λ* = 0.154 nm). The camera length was 210 mm, and measurements were conducted at 30 °C. UV–VIS spectroscopy was performed using a V-670 spectrophotometer (JASCO, Tokyo, Japan) at a scan speed of 400 nm/min over a wavelength range of 200–800 nm at room temperature. Transmittance was calculated using Equation (3) with a thickness correction of 0.02 cm to account for sample dimensions.

*T*_1_ = exp (−*μt*_1_) (1)

*T*_2_ = exp (−*μt*_2_) (2)

*T*_1_ = exp (In(*T*_2_/100) × (*t*_1_/*t*_2_)) × 100 (3)

*T*_1_ = Transmittance after thickness correction (%), *T*_2_ = Measured transmittance (%), *t*_1_ = Thickness correction value = 0.02 (cm), *t*_2_ = Thickness of sample (cm), *μ* = Linear absorption coefficient. A multi-wavelength Abbe refractometer (DR-M4/1550; ATAGO, Tokyo, Japan) was used. The measurement temperature was maintained at room temperature, and methylene iodide (*n*_D20_ = 1.74) was used as the intermediate standard solution. Note that *n*_D_ represents the refractive index under a sodium light source (wavelength *λ* = 589 nm).

### 2.5. SAXS Data Analysis Procedure

#### 2.5.1. Ruland Correction and Background Subtraction

To eliminate thermal diffuse scattering from the SAXS profiles, the background contribution was approximated using the Ruland empirical function [27,28]:

*I*_bg_(*q*) = A*q*^−2^ + B (4)

where *q* is the scattering vector, and A and B are fitting constants. The fitting was performed in the high-*q* region (3.5–4.5 nm^−1^), where the scattering intensity of PDLLA exhibits a monotonic decay without structural features. The obtained *I*_bg_(*q*) was subtracted from the experimental data to yield the Ruland-corrected SAXS intensity:

*I*_corr_(*q*) = *I*_exp_(*q*) − *I*_bg_(*q*) (5)

The background fitting for neat PDLLA produced satisfactory results (*R*^2^ = 0.918), as summarized in Table S1, confirming the reliability of the correction procedure (Figure S1). To isolate the scattering contribution of the silica domains, the Ruland-corrected SAXS profile of neat PDLLA was subtracted from those of the hybrid films:

*I*_hybrid–silica_(*q*) = *I*_corr,hybrid_(*q*) − *I*_corr,PDLLA_(*q*) (6)

This subtraction was limited to the low-*q* region (0.1–3.0 nm^−1^), where the Ruland correction is valid and the scattering intensity primarily reflects silica domain correlations rather than the polymer thermal background.

#### 2.5.2. Transformation into Electron Density Correlation Function *γ*(*r*)

The corrected scattering intensities were transformed into the electron density correlation function *γ*(*r*) to extract characteristic structural distances in the hybrids. Transformation was performed using a sine transform, which is mathematically equivalent to the isotropic inverse Fourier transform for SAXS [29].

(7) $$\gamma \left(r\right)=\int_{0}^{\infty }I\left(q\right)\cdot \frac{\sin qr}{qr}\;dq$$

Constant prefactors were omitted, and the resulting *γ*(*r*) was normalized. This approach assumes spherical symmetry and corresponds to a one-dimensional Hankel-type transform. No window functions or extrapolation were applied; instead, numerical integration was performed directly over the experimental *q*-range, followed by normalization.

#### 2.5.3. Porod Analysis

In the high-*q* region (0.3–0.8 nm^−1^), the Porod law was applied to evaluate interfacial characteristics [30,31]:

*I*(*q*) ∝ *q*^−m^ (8)

where m denotes the Porod exponent. The fitted curves are shown in Figure S2 and Table S2.

## 3. Results and Discussion

### 3.1. Characterization of PDLLA Hybrid Films

**Figure 1:**
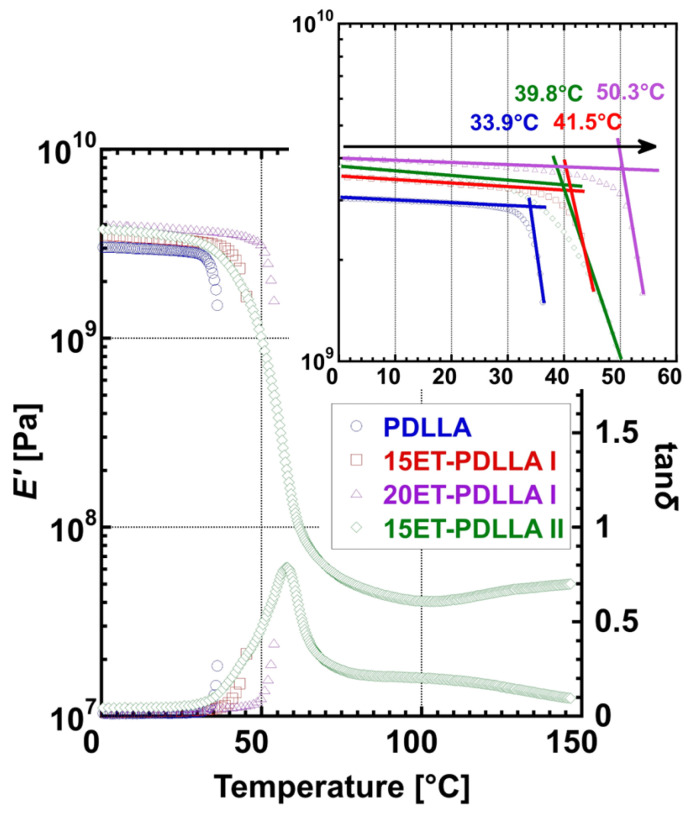
DMA properties of PDLLA, PDLLA–I and –II/SiO_2_ hybrid films.

Hybrid films were prepared via the sol–gel method using ET-PDLLA I and TEOS. TEOS was incorporated at 15 wt% (15ET-PDLLA I) and 20 wt% (20ET-PDLLA I), as transparency decreased significantly above 30 wt% loading. For comparison, a film of ET-PDLLA II containing 15 wt% TEOS (15ET-PDLLA II) was also prepared. Dynamic mechanical analysis (DMA) was used to evaluate the thermomechanical properties (Figure 1). The 15ET-PDLLA II film formed a 3D network due to the presence of silane groups at both chain ends, as indicated by a plateau region above 80 °C [14,16,17]. In contrast, PDLLA, 15ET-PDLLA I, and 20ET-PDLLA I films exhibited no plateau, confirming that they adopted graft-type rather than network-type structures. The onset temperatures of the storage modulus (*E*′) were 34 °C for neat PDLLA, 41.5 °C for 15ET-PDLLA I, and 50 °C for 20ET-PDLLA I, demonstrating a progressive increase with silica loading. This trend indicates that polymer chain mobility was reduced by interactions between PDLLA and the grafted silica. Furthermore, the inflection temperature of *E*′ was similar for 15ET-PDLLA II (40 °C) and 15ET-PDLLA I, suggesting that the glass transition behavior is primarily governed by silica content rather than structural type.

**Figure 2:**
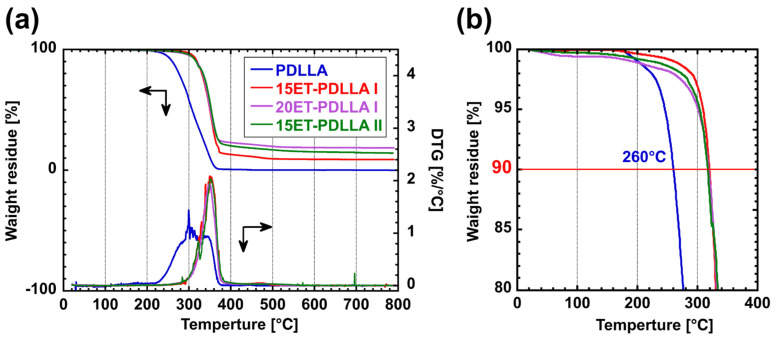
(**a**) TGA curves of PDLLA, PDLLA–I and –II/SiO_2_ hybrid films. (**b**) Enlarged view showing the 10 wt% weight loss temperature.

TGA was used to determine the silica content based on the residual weight at 800 °C. The measured values were 8.8 wt% for 15ET-PDLLA I, 18.5 wt% for 20ET-PDLLA I, and 11.7 wt% for 15ET-PDLLA II. The higher residue in 15ET-PDLLA II compared with 15ET-PDLLA I can be attributed to the additional alkoxysilane groups at both ends of ET-PDLLA II (Figure 2a). These measured residues were also consistent with theoretical values, assuming complete conversion of terminal silanol groups to silica. To further clarify thermal degradation behavior, DTG (d*m*/d*T*) curves were analyzed alongside the TGA data. Neat PDLLA exhibited a broad DTG peak over a wide temperature range, reflecting its fully amorphous, stereoirregular chain structure, broad molecular weight distribution, and multiple degradation pathways associated with reactive end groups. In contrast, all hybrid films showed sharp, well-defined DTG maxima, indicating that silica incorporation promotes a more uniform degradation process by restricting chain mobility and suppressing competing scission and unzipping reactions near the polymer–silica interface. Thermal resistance was further evaluated by comparing the 10% weight-loss temperatures (Figure 2b). Neat PDLLA decomposed at 260 °C, whereas the hybrid films exhibited significantly higher decomposition temperatures: 319 °C (15ET-PDLLA I), 318 °C (20ET-PDLLA I), and 321 °C (15ET-PDLLA II), corresponding to improvements of approximately 60 °C.

DMA and TGA data are presented in Table 1. Interestingly, no significant difference in thermal stability was observed between grafted and networked hybrids, despite their distinct structural motifs. This similarity can be explained by the fundamental role of nanoscale silica domains in controlling degradation and chain dynamics. In both architectures, silica incorporation introduces abundant polymer–silica interfaces that restrict the local mobility of PDLLA chains through hydrogen bonding and dipole–dipole interactions. Once the interfacial volume fraction exceeds a threshold, the extent of chain confinement is primarily determined by the overall silica content rather than the connectivity of the domains. In the network-type hybrid, chain segments are confined by covalent linkages at both chain ends, whereas in the graft-type hybrid, chains are tethered at one end but interact noncovalently with neighboring domains. Although the spatial distribution differs, both structures effectively suppress segmental relaxation and retard thermal decomposition by creating an interphase region with reduced free volume and hindered diffusion of degradation products.

Moreover, the barrier effect of silica—manifested as reduced oxygen permeability and delayed volatilization of decomposition fragments—occurs independently of network topology [32,33,34,35]. Previous studies have shown that the efficiency of this barrier effect depends on particle dispersion and interfacial adhesion rather than the connectivity of silica clusters. Consequently, the nearly identical thermal stability of grafted and networked hybrids highlights that silica loading and interfacial interactions govern the thermal response, while structural type primarily influences mesoscopic mechanical behavior (e.g., plateau formation in DMA). In this context, the modest structural dependence observed here indicates that, although grafted and networked architectures differ in mesoscale connectivity, both generate a comparable level of polymer–silica interfacial confinement once the silica content exceeds a critical fraction. Because the specific interfacial area S_v_ is predominantly determined by silica loading rather than architectural topology, both hybrids achieve a similar degree of constrained polymer dynamics with respect to thermal degradation, even though their mechanical responses remain distinct.

### 3.2. SAXS Profiles of PDLLA Hybrid Films

**Figure 3:**
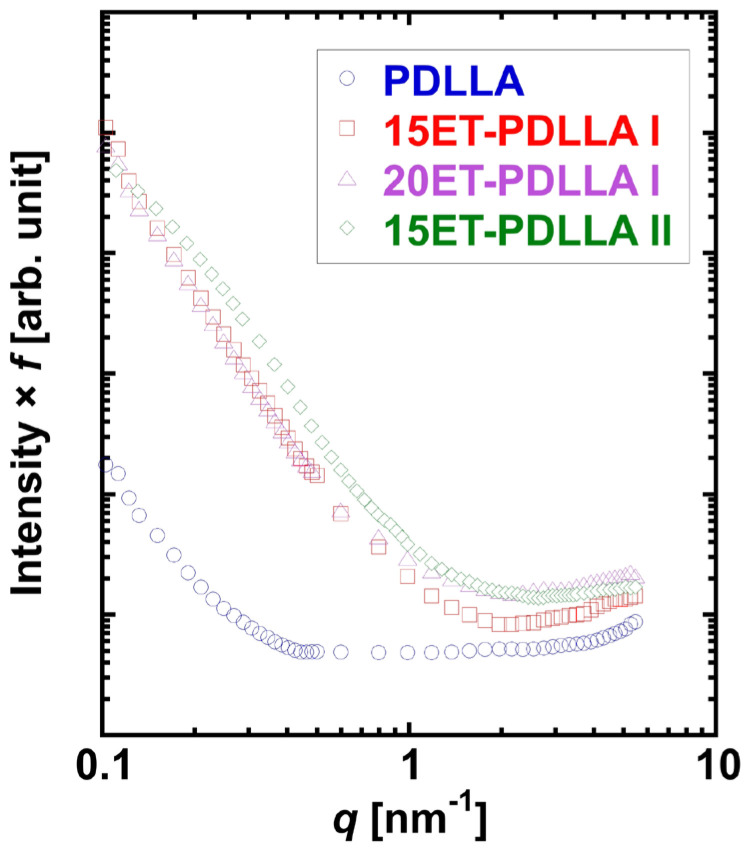
SAXS profiles of neat PDLLA and ET–PDLLA hybrid films with different silica contents.

Structural analysis is essential to understand how the nanoscale organization of silica domains underlies the observed thermal and thermomechanical behaviors. Because thermal degradation is governed by interfacial interactions, whereas mechanical relaxation is dictated by mesoscale connectivity, small-angle X-ray scattering (SAXS) provides a direct means to assess whether the spatial arrangement of silica differs between graft-type and network-type hybrids. In particular, variations in domain spacing, inter-domain correlations, or the emergence of network-like features are expected to influence the DMA response but have minimal impact on TGA/DTG behavior. To examine these structural signatures, SAXS profiles of the neat polymer and hybrid films were analyzed (Figure 3). All samples exhibited monotonically decreasing intensities with increasing *q*, without distinct peaks, indicating that the silica nanoparticles were amorphous and uniformly dispersed in the polymer matrix. Compared with neat PDLLA, the hybrid films displayed markedly higher scattering intensities in the low-*q* region, consistent with the presence of silica domains. For ET-PDLLA I, the overall intensity systematically increased with silica content from 8.8 wt% to 18.5 wt%, reflecting enhanced particle–particle correlations at higher loadings. In contrast, the ET-PDLLA II hybrid (11.7 wt%) showed a similar global profile but with slightly elevated intensity at intermediate *q*, suggesting a tendency toward a three-dimensional network structure.

**Figure 4:**
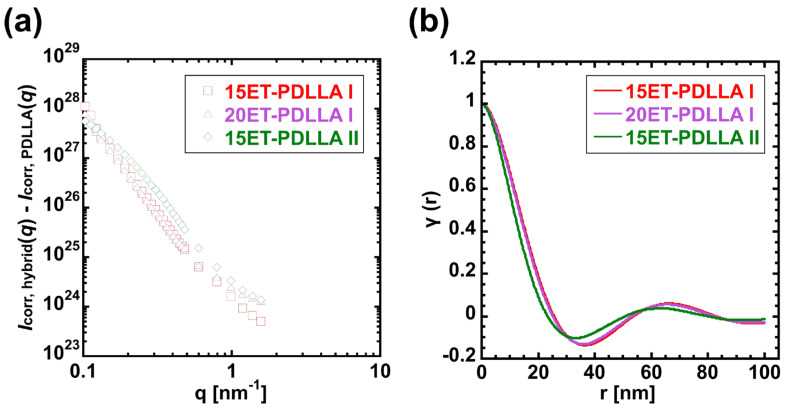
(**a**) Corrected SAXS profiles of ET–PDLLA hybrid films after subtraction of the Ruland background of neat PDLLA. (**b**) Corresponding electron density correlation functions *γ*(*r*) obtained by isotropic sine transformation.

Figure 4a presents the SAXS profiles after subtraction of the Ruland background of neat PDLLA. Removal of the monotonic high-*q* tail, arising from thermal density fluctuations of the polymer matrix, effectively isolated the scattering contribution of the silica domains. After correction, the hybrids retained enhanced low-*q* intensity relative to PDLLA, confirming domain-level correlations attributable to silica aggregation. To extract characteristic structural distances, the corrected SAXS intensities were transformed into the electron density correlation function *γ*(*r*) via an isotropic sine transform (Figure 4b). The first minimum followed by the first maximum was assigned to the inter-domain distance *R*_d_. The values were *R*_d_ ≈ 66.2 nm (8.8 wt%), 65.6 nm (18.5 wt%), and 64.0 nm (11.7 wt%), yielding an average of *R*_d_ = 65.2 ± 1.1 nm. The weak dependence of *R*_d_ on silica loading indicates that the characteristic spacing between silica domains is primarily governed by the sol–gel growth and packing process rather than by silica content. Meanwhile, the correlation strength, reflected in the amplitude of *γ*(*r*), increased with loading, consistent with stronger interparticle interactions. For comparison, the end-to-end distance of the PDLLA chain was estimated using the Gaussian relation *R*_0_ = 0.0868 M^1/2^ [nm], yielding *R*_0_ ≈ 24–30 nm for *M*_w_ = 7.5–12.0 × 10^4^ g/mol. The effective domain size of silica can thus be approximated as *R*_d_ − *R*_0_, giving 30–35 nm. This provides a structural rationale for the possibility of chain bridging and entanglement, which stabilizes the hybrid microstructure irrespective of the silica loading level. Importantly, the nearly constant *R*_d_ values demonstrate that domain coarsening does not occur, even at higher silica concentrations.

In the high-*q* region (0.3–0.8 nm^−1^), Porod analysis was performed according to *I*(*q*) ∝ *q*^−*m*^. The graft-type hybrids (ET-PDLLA I) exhibited smaller Porod exponents (*m* ≈ 3.2–3.9), reflecting diffuse polymer–silica interfaces and dominant interparticle correlations. In this case, the silica particles were predominantly dispersed in an isolated manner, with the interfacial regions broadened by the surrounding polymer chains. Consequently, the interdomain spacing remained constant, but the connectivity between the domains was weak, preventing the development of a continuous network. By contrast, the network-type ET-PDLLA II showed a larger exponent (*m* ≈ 3.9), close to the Porod limit (*m* → 4), indicating smoother effective interfaces and more spatially correlated, fractal-like silica arrangements. This can be attributed to the presence of silane groups at both chain ends, which facilitated interdomain bridging and promoted the formation of a three-dimensional silica network. Such a 3D network extends beyond local particle–particle correlations, enhancing domain connectivity at the mesoscale. Notably, the increase in the Porod exponent toward ~4 directly correlates with smoother and more cohesive interfaces, which explains the plateau behavior of ET-PDLLA II in DMA. This demonstrates that once the interfacial volume fraction of silica exceeds a certain threshold, the system transitions from isolated particle confinement to cooperative network-induced chain restriction.

To quantitatively interpret the chain confinement behavior, the interfacial parameters governing polymer mobility were calculated by combining the SAXS-derived structural parameters with the TGA-derived silica fractions. The silica mass fraction was first obtained from the TGA residue, corresponding to the inorganic content remaining after complete thermal decomposition of PDLLA. This mass fraction was converted into a volume basis using the densities of silica (*ρ*_s_ = 2.0 g/cm^3^) and PDLLA (*ρ*_p_ = 1.25 g/cm^3^):

*ϕ*_s_ = (*w*_s_/*ρ*_s_)/((*w*_s_/*ρ*_s_) + (*w*_p_/*ρ*_p_)) (9)

yielding silica volume fractions of *ϕ*_s_ = 0.055, 0.073, and 0.116 for the 8.8, 11.7, and 18.5 wt% samples, respectively.

The center-to-center distance between silica domains (*R*_d_) was obtained from the primary SAXS peak. Assuming a simple cubic packing model, the effective silica domain diameter is calculated as follows:

*D* = *R*_d_ (6*ϕ*_s_/π)^1/3^ (10)

with domain sizes of approximately 28, 31, and 37 nm, respectively, which agreed well with the particle sizes estimated from the Guinier radius *R*_0_.

The specific interfacial area per unit composite volume was then calculated as:

*S*_v_ = 6*ϕ*_s_/*D* (11)

resulting in *S*_v_ = 1.18 × 10^7^, 1.41 × 10^7^, and 1.88 × 10^7^ m^2^/m^3^ for the 8.8, 11.7, and 18.5 wt% samples, respectively. These values indicate that the available polymer–silica interfacial area increases by a factor of approximately 1.6 between the lowest and highest silica loadings.

Assuming an interphase thickness *δ*, the interfacial volume fraction was evaluated as:

*ϕ*_int_ = *S*_v_ *δ* (12)

where *ϕ*_int_ represents the fraction of polymer segments located within the restricted-mobility region formed around the silica surface. A larger *ϕ*_int_ corresponds to a greater proportion of chain segments experiencing suppressed mobility due to polymer–silica interactions.

These interfacial parameters provide a quantitative structural rationale for the thermomechanical and thermal behaviors described in Section 3.1. The increase in *S*_v_ from 15ET-PDLLA to 20ET-PDLLA reflects a substantial increase in the number of interfacially confined chains, which is consistent with the upward shift in the storage modulus onset temperature observed by DMA. In contrast, the 10% weight-loss temperature in the TGA increased only slightly because the initial decomposition was governed by bond scission chemistry, which was not strongly affected by interfacial confinement. Overall, the SAXS-derived parameters *R*_d_, *D*, and *S*_v_, together with the resulting interfacial volume fraction *ϕ*_int_, coherently account for the enhanced chain confinement and the improved thermomechanical stability of the PDLLA–silica hybrids.

### 3.3. Optical Properties of PDLLA Hybrid Films

**Figure 5:**
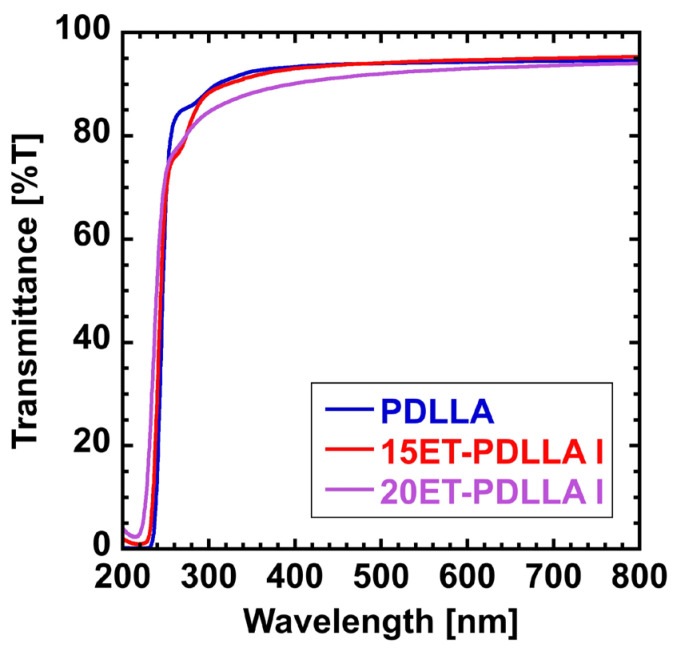
UV-vis spectra of PDLLA, PDLLA–I/SiO_2_ hybrid films.

Figure 5 shows the UV–Vis spectra of PDLLA and the hybrid films normalized to a thickness of 200 μm. All the hybrid films with up to 18 wt% silica exhibited high visible light transmittance (>90%). The transmittance at 400 nm decreased only slightly from 93% (15ET-PDLLA I) to 90% (20ET-PDLLA I), despite the silica content more than doubling. This behavior indicated that the silica domains remained uniformly dispersed within the Rayleigh scattering regime (domain diameter < 40 nm), thereby suppressing wavelength-scale scattering even at high loadings. In addition, no domain coarsening was observed in SAXS, which is crucial for maintaining optical transparency. However, above 25 wt% TEOS, the transparency decreased sharply. Based on SAXS-derived structural parameters, this loss of transparency is attributed not to particle growth but to a reduction in the inter-domain spacing. As silica volume fraction increases, the particle–particle distance becomes sufficiently small that domain–domain correlations generate refractive-index fluctuations at length scales comparable to visible wavelengths, resulting in increased scattering. Thus, ~18 wt% represents the practical upper limit for maintaining transparency while preserving nanoscale particle separation.

**Figure 6:**
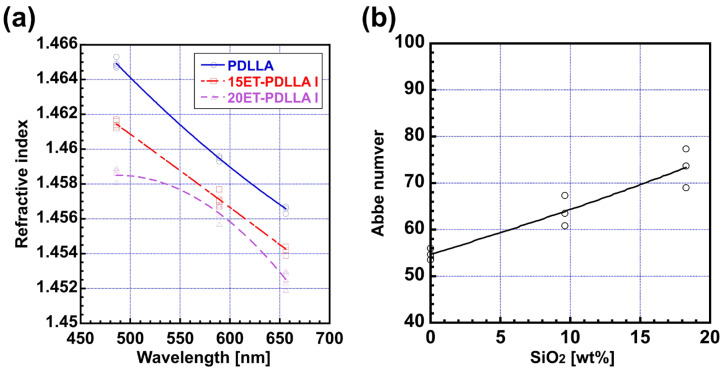
(**a**) Refractive index at each wavelength of PDLLA, PDLLA–I/SiO_2_ hybrid films (**b**) Changes in silica content and Abbe number of grafted PDLLA.

The refractive-index spectra are shown in Figure 6a. The refractive index decreased across all wavelengths as the silica content increased owing to the amorphous nature of silica and its intrinsically lower refractive index compared to that of PDLLA. From these data, the Abbe number was calculated as a function of silica content (Figure 6b). The Abbe number increased progressively with silica loading, from 55 for neat PDLLA to 73 for 20ET-PDLLAI, representing an enhancement of 18 units. This improvement indicates that the addition of silica reduces chromatic dispersion and enhances optical uniformity throughout the material.

Table 2 summarizes the quantitative optical parameters, including the transmittance at 400 nm (%T), refractive index at the D-line (*n*_D_), and Abbe number (*V*_D_), each reported with standard deviations. The observed optical trends can be rationalized using the structural information obtained in Section 3.2. SAXS revealed that the characteristic interdomain spacing (~65 nm) was nearly independent of silica loading, and the silica domain diameter remained within the Rayleigh regime. Consequently, the scattering loss remained minimal for up to 18 wt% silica. In contrast, the interfacial analysis showed that the specific interfacial area (*S*_v_ = 6*ϕ*_s_/*D*) increases monotonically with silica loading. Although *S*_v_ does not directly determine the optical transparency, it reflects the fraction of polymer chains located in interfacial regions with restricted mobility. DMA confirmed that chain mobility decreases with silica loading, which is consistent with a larger interfacial volume fraction (*ϕ*_int_ = *S*_v_ *δ*). Such mobility suppression reduces the local density fluctuations in the polymer matrix, contributing to a more homogeneous refractive-index distribution. Because the refractive index is directly correlated with the local density through the Lorentz–Lorenz relation, the suppression of density fluctuations leads to smaller spatial variations in the refractive index, thereby reducing the chromatic dispersion. Consequently, the Abbe number increases with increasing silica content. In summary, the high transparency of hybrid films with up to 18 wt% silica is primarily governed by the nanoscale size and uniform dispersion of the silica domains, which minimizes Rayleigh scattering. The simultaneous increase in the Abbe number was attributed to the enhanced refractive-index uniformity arising from the increased interfacial polymer confinement. These combined structural and interfacial effects provide a coherent explanation for the optical behavior of PDLLA–silica hybrid films.

## 4. Conclusions

Organosilane-terminated PDLLA was successfully synthesized and used to prepare transparent PDLLA/silica hybrid films via a sol–gel reaction with TEOS without phase separation, enabling systematic evaluation of how silica incorporation influences thermomechanical, thermal, and optical properties. Quantitative characterization showed that the onset of the storage modulus increased from 34 °C for neat PDLLA to 50 °C at 20 wt% silica, while the 10% weight-loss temperature improved by ~60 °C, reflecting the stabilizing effect of polymer–silica interfacial interactions. SAXS measurements revealed inter-domain distances of ~60–65 nm and effective silica domain sizes of 30–35 nm, which, together with the increasing specific interfacial area, clarified how nanoscale dispersion governs chain confinement. Importantly, the small and uniformly dispersed silica domains remained within the Rayleigh regime, enabling >90% optical transmittance up to 18 wt% loading and promoting refractive-index homogeneity that increased the Abbe number from 55 to 73. These results advance the understanding of PDLLA/silica systems by demonstrating how nanoscale structure—not only silica content but also domain size and interfacial volume—governs optical performance, complementing earlier work that focused primarily on mechanical reinforcement. The hybrid films highlight the potential of biodegradable PDLLA as a sustainable optical-material platform, although long-term durability, environmental aging, and mechanical robustness under cyclic stress remain important areas for future study. Overall, this study provides quantitative insight into the structure–property relationships that enable high transparency, enhanced thermal stability, and improved optical behavior in PDLLA–silica hybrids.

## Figures and Tables

**Table 1:** Thermal and mechanical properties of PDLLA and ET–PDLLA hybrid films obtained from DMA and TGA analyses.

Sample	*T*g by *E*′ Onset [°C]	*E*′_25°C_ [Pa]	*E*′*_T_*_g+25°C_ [Pa]	*T*_10%_ [°C]
PDLLA	33.9	2.90 × 10^9^	-	259.5
15ET-PDLLA I	41.5	3.31 × 10^9^	-	319.7
20ET-PDLLA I	39.8	3.83 × 10^9^	-	318.2
15ET-PDLLA II	50.3	3.58 × 10^9^	6.92 × 10^7^	317.8

**Table 2:** Transmittance at 400 nm, refractive indices (*n* ± *SD*) measured at wavelengths of 486 nm, 589 nm, and 656 nm, and Abbe number (*V_D_* ± *SD*) of PDLLA–based films.

Sample	%T_400nm_	*n*_486_ ± *SD*	*n*_589_ ± *SD*	*n*_656_ ± *SD*	*V_D_* ± *SD*
PDLLA	93.41	1.465 ± 2.3 × 10^−4^	1.460 ± 1.1 × 10^−4^	1.457 ± 1.6 × 10^−4^	54.7 ± 1.3
15ET-PDLLA I	90.17	1.461 ± 2.1 × 10^−4^	1.457 ± 3.6 × 10^−4^	1.454 ± 2.2 × 10^−4^	63.9 ± 0.3
20ET-PDLLA I	93.05	1.459 ± 2.4 × 10^−3^	1.456 ± 1.3 × 10^−3^	1.453 ± 7.1 × 10^−4^	73.3 ± 4.1

## Data Availability

The raw data supporting the conclusions of this article will be made available by the authors on request.

## Supplementary Materials

The following supporting information can be downloaded at: https://www.mdpi.com/article/10.3390/polym17233202/s1, Figure S1: SAXS profiles of PDLLA and hybrid films before and after Ruland correction. The dashed line indicates the fitted background, and the solid lines show the corrected intensities; Figure S2: Porod fitting curves of SAXS data for hybrid films with various silica contents; Table S1: Fitting parameters for the Ruland correction of PDLLA; Table S2: Fitting parameters obtained from the Porod analysis of hybrid films.
