# Supplementary material for: Structure–Property Relationships in PDLLA/Silica Hybrid Films: Impact of Grafting and Network Formation on Optical Behavior
Source: Polymers (Basel). 2025 Nov 30;17(23):3202. doi: 10.3390/polym17233202 (PMC12694312; doi:10.3390/polym17233202)
Supplement: Supplementary file 1 [file polymers-17-03202-s001.zip › polymers-3999034-supplementary.pdf]

Characterization of PDLLA grafted onto nano silica and effect of their structure for optical properties.

Shuta Hara[1], Keiya Kawamura[2], Atsushi Furukawa[2], Shigeru Shimizu[2], Hiroki Ikake*[2]

1: Department of Material and Life Chemistry, Kanagawa University, 3-6-1, Kanagawa-ku, Yokohama 221-8686, Japan

2: Department of Materials and Applied Chemistry, College of Science and Technology, Nihon University, 1-8-14 Kandasurugadai, Chiyoda-ku, Tokyo 101-8308, Japan Email: ikake.hiroki@nihon-u.ac.jp

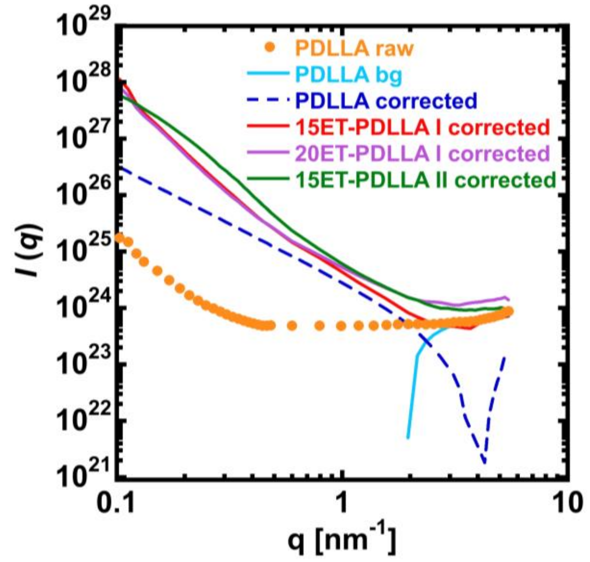

Figure S1 SAXS profiles of PDLLA and hybrid films before and after Ruland correction. The dashed line indicates the fitted background, and the solid lines show the corrected intensities.

Table S1. Fitting parameters for the Ruland correction of PDLLA.

**q-range [nm⁻¹]**	**n (data points)**	**A (±SE) [arb. units]**	**B (±SE) [arb. units]**	**SSE**	**MSE**	**RMSE**	**R^2**
**3.5 – 4.5**	**6**	**$-3.10\times10^{24} \pm 0.46\times10^{24}$**	**$8.12\times10^{23} \pm 0.30\times10^{23}$**	**5.90×10^{44}**	**1.48×10^{44}**	**1.21×10^{22}**	**0.918**

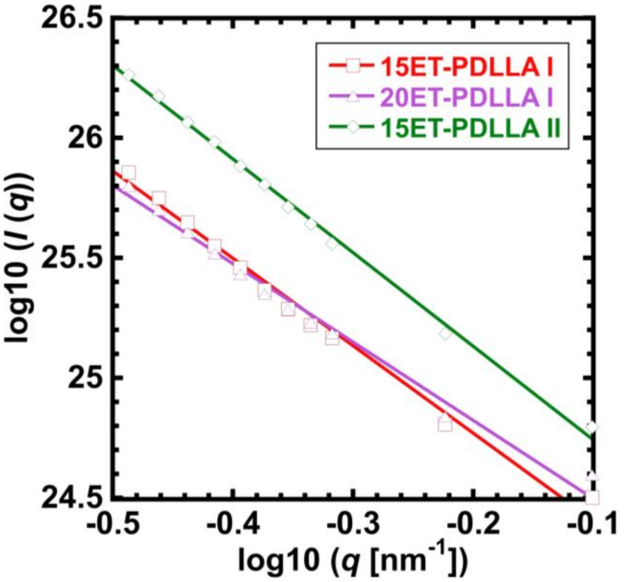

Figure S2 Porod fitting curves of SAXS data for hybrid films with various silica contents.

Tabel S2 Fitting parameters obtained from the Porod analysis of hybrid films.

	m	R^2
8.8wt%-I silica	-3.643	0.988
18.5wt%-I silica	-3.265	0.983
11.7wt%-II silica	-3.900	0.997
